# GC/MS Analysis, Cytotoxicity, and Antimicrobial Properties of Six Moroccan Essential Oils Traditionally Used for COVID-19 Prevention

**DOI:** 10.3390/molecules30214179

**Published:** 2025-10-25

**Authors:** Houda Zaher, José Francisco Quílez del Moral, Sanae Lemrabet, Azucena González-Coloma, Bouchaib Bencharki

**Affiliations:** 1 Laboratory of Agro Alimentary and Health, Faculty of Sciences and Techniques, Hassan First University of Settat, B.P. 539, Settat 26000, Morocco; bouchaib.bencharki@uhp.ac.ma; 2 Department of Organic Chemistry, Institute of Biotechnology, University of Granada, 18071 Granada, Spain; 3 Virology Department, National Institute of Hygiene, Ministry of Health, B.P. 769, Rabat 10020, Morocco; sanae.lem@gmail.com; 4 Institute of Agricultural Sciences, Spanish National Research Council (CSIC), 28006 Madrid, Spain

**Keywords:** phytochemicals, antimicrobial activity, cytotoxicity, essential oils, Moroccan medicinal plants, COVID-19 prevention

## Abstract

The COVID-19 pandemic has reignited interest in traditional medicinal plants as potential therapeutic agents. This study examined the chemical composition, cytotoxicity, and antimicrobial activity of essential oils from six Moroccan medicinal plants, namely, *Eucalyptus globulus*, *Artemisia absinthium*, *Syzygium aromaticum*, *Thymus vulgaris*, *Artemisia alba*, and *Santolina chamaecyparissus*, which are commonly used by the Moroccan population for COVID-19 prevention. The chemical composition of each essential oil was determined using gas chromatography–mass spectrometry (GC–MS) to identify key compounds. Cytotoxicity was evaluated in the Vero E6 cell line, which is frequently used in SARS-CoV-2 research, using the neutral red assay, with oil concentrations ranging from 25 to 100 µg/mL. Antimicrobial activity was tested against standard reference strains, including *Escherichia coli* (ATCC 25922), *Pseudomonas aeruginosa* (ATCC 27853), *Staphylococcus aureus* (ATCC 25923), *Candida albicans* (ATCC 10231), and *Bacillus subtilis* (ATCC 6633), using the disc diffusion method. GC–MS analysis revealed significant components such as spathulenol (15%) and caryophyllene oxide (7.67%) in *Eucalyptus globulus* and eugenol (54.96%) in *Syzygium aromaticum*. Cytotoxicity assays indicated that higher concentrations of essential oils significantly reduced cell viability, with *Thymus vulgaris* showing the highest IC_50_ (8.324 µM) and *Artemisia absinthium* the lowest (18.49 µM). In terms of antimicrobial activity, *Eucalyptus globulus* had the strongest effect, with a 20 ± 0.00 mm inhibition zone against *Bacillus subtilis*, whereas both *Syzygium aromaticum* and *Artemisia herba-alba* had a 12.25 ± 0.1 mm inhibition zone against the same strain. These findings suggest that these essential oils have significant therapeutic potential, particularly in combating antimicrobial resistance and exerting cytotoxic effects on viral cell lines. Further research is necessary to explore their mechanisms of action and ensure their safety for therapeutic use.

## 1. Introduction

Since the Middle Ages, aromatherapy has been employed in traditional pharmacopoeia for a wide range of applications, including cancer treatment; anti-inflammatory, antibacterial, and antiviral activities; and insecticidal, medicinal, and cosmetic uses [1]. The essential oils derived from these plants contain a complex mixture of volatile compounds, such as monoterpenes, sesquiterpenes, phenolic derivatives, and aliphatic compounds, which are believed to contribute to their therapeutic effects [2]. The chemical composition of these oils can vary significantly depending on the plant species, geographic origin, and extraction methods [2]. To date, the importance of aromatic oils remains significant, with an increased understanding of their mechanisms of action on various biological and medicinal activities, such as their cytotoxic and antimicrobial effects.

Like many other countries, Morocco has a long history of the use of aromatic medicinal plants for curative and preventive purposes. Its Mediterranean location and diverse climatic conditions enrich its vegetation and plant species. In addition, the herbal market in Morocco has good knowledge of the benefits and medicinal uses of plants, which means that the majority of Moroccans consumed different plants at the height of the COVID-19 pandemic, not only because of the lack of other treatments but also because they are less expensive and more available [3]. Moreover, numerous ethnobotanical studies conducted during the pandemic in all of Morrocco’s prefectures provide valuable insights into traditional knowledge and practices related to the use of these plants, serving as a basis for scientific investigation. The aromatic medicinal plants examined in this study, including *Thymus vulgaris* L., *Eucalyptus globulus*, and *Artemisia absinthium* L., are among the most widely distributed and used by the Moroccan population for treating respiratory ailments. For example, *Artemisia absinthium* and *Thymus vulgaris* L. are commonly consumed as tea by Moroccans during the winter season. On the other hand, cytotoxic effects were observed in vitro against bacteria and eukaryotic cells, causing membrane damage, depolarization of mitochondrial membranes by decreasing the membrane potential, coagulation of the cytoplasm, and damage to cellular lipids and proteins, as well as exerting prooxidant activity [1]. These cytotoxic properties can disrupt the cellular function of microorganisms, making essential oils effective as antiseptic and antimicrobial agents for personal hygiene and air purification [1]. This attribute makes essential oils promising candidates for further research on multidrug-resistant bacteria. Indeed, aromatic plants have demonstrated notable activity in various studies [4,5,6,7,8,9,10,11]. The rise of multidrug resistance, combined with the adverse effects of current antibiotics on public health and the lack of new effective antibiotics, underscores the urgent need to explore alternative treatments [12].

Currently, the COVID-19 pandemic, caused by the novel coronavirus SARS-CoV-2, has highlighted the urgent need for effective treatments and preventive measures. Various studies have reported the promising anti-COVID-19 properties of essential oils from plants such as *Mentha pulegium*, *Mentha microphylla*, *Mentha vilosa*, *Mentha thymifolia*, *Illicium verum*, *Syzygium aromaticum*, *Citrus limon*, and *Pelargonium graveolens*. These essential oils have demonstrated a selectivity index (SI) of more than four against SARS-CoV-2 [13]. The vaporous nature of essential oils gives them an advantage in targeting the respiratory system, where SARS-CoV-2 primarily infects. Eucalyptol, for example, has been shown to have a direct virucidal effect by interfering with the binding of SARS-CoV-2 spike proteins to ACE2 receptors. This compound tends to concentrate in the lungs, particularly in the lower respiratory tract, where it may exert a local virucidal effect [14,15].

The cytotoxicity of essential oils may correlate with their antiviral activity, as previous research has demonstrated a direct relationship between high cytotoxicity and strong anti-SARS-CoV-2 effects. For example, essential oils such as *Syzygium aromaticum*, *Cymbopogon citratus*, *Citrus limon*, *Pelargonium graveolens*, *Origanum vulgare*, and *Matricaria recutita* exhibited high antiviral activity, which was accompanied by high cytotoxicity. In contrast, *Zingiber officinalis*, *Melaleuca alternifolia*, and *Rosmarinus officinalis* showed lower cytotoxicity and, correspondingly, lower anti-SARS-CoV-2 activity [16].

This study offers new insights into the chemical composition, cytotoxicity, and antimicrobial activity of essential oils from Moroccan medicinal plants, including *Artemisia absinthium* L., *Syzygium aromaticum*, *Artemisia herba-alba*, *Eucalyptus globulus*, *Thymus vulgaris* L., and *Santolina chamaecyparissus*. Although these plants have been investigated in prior research,

These plants species have received considerable attention, and numerous biological activities have been reported for the corresponding essential oils. A noteworthy difference in the chemical composition of oils collected in different geographical areas emerges from the analysis of these studies [17,18,19,20,21,22,23]. Regarding *E. globulus*, the monoterpene 1,8-cineole (30%), is usually identified as the major components of its essential oil [24]. *A. absithium* is probably the species presenting more variability in the chemical composition of its essential oils, although β-thujone and *trans*-sabinyl acetate are recognized as major components of its essential oil [25]. Eugenol is the major compound of clove (*S. aromaticum*) essential oil, accounting for at least 50% [26]. composition. The major component of thyme essential oil is thymol, although the presence of *p*-cymene is also significant in this essential oil [27]. *A. alba* is characterized by a high content of camphor [28]. Finally, artemisia ketone is generally the major constituent identified in the essential oil of *S. chamaecyparissus* [29].

In addition to the remarkable number of biological applications attributed to these essential oils, three of them, namely *E. globulus*, *S. aromaticum* and *T. vulgaris*, have been evaluated for their potential anti-coronavirus disease 2019 (COVID-19) effects [30].

The observed strong geographic influence on the chemical composition of the essential oils, as well as the evidence of their potential activity against COVID-19, led us to conduct this study. Thus, by identifying their chemical compounds and establishing noncytotoxic concentrations, we provide valuable data that can guide further exploration of these essential oils for potential antiviral or anti-COVID-19 applications. Notably, this research included *Artemisia absinthium* L., *Santolina chamaecyparissus*, and *Artemisia herba alba*, which were tested on the Vero E6 cell line for the first time, offering essential insights into the safe and effective use of these oils in therapeutic contexts.

## 2. Results and Discussion

### 2.1. Chemical Composition of the Essential Oils

The essential oil yields obtained from the studied plants ranged from 0.3% to 7.5% (*v*/*w*), with *S. aromaticum* showing the highest yield at approximately 7.5%, followed by *E. globulus* (3.0%), *T. vulgaris* (2.0%), *A. absinthium* (1.2%), *S. chamaecyparissus* (0.8%), and *A. herba-alba* (0.5%). Variations in yield can be attributed to differences in plant material, harvesting conditions, and extraction techniques.

The complexity of the essential oils was identified and quantified by chromatography coupled with mass spectrometry (GC–MS) analysis. Our findings revealed differences from those of previous analyses, highlighting variations in the chemical profiles and potential implications for their use.

In the absinthe oil analyzed, 51 compounds were identified, representing 99.81% of the total compounds. The results revealed that the oil is rich in α-thujone (**3**) (29.02%) and camphor (**4**) (24.34%), with notable amounts of chamazulene (6.92%) and (−)-4-terpineol (3.68%) (Table 1).

As previously stated, in most of the previous studies, the presence of the β-thujone isomer has been usually reported to be greater than that of its isomer α-thujone (**3**) in the oil [31]. Contrary to expectations, our study revealed that α-thujone (**3**) was present at a remarkably high level of 29%, whereas β-thujone was detected at only 1.67%. Surprisingly, another Moroccan study revealed that *A. absinthium* essential oil was rich in 3,3,5-trimethylcyclohexene (27.93%), which was absent in our study, and camphor (**4**) (22.50%, a level similar to what we found), but no α-thujone or β-thujone were detected in the sample [22]. At least 17 major compounds, including myrcene, sabinene, sabinyl acetate, epoxyocimene, chrysanthenol, chrysanthenyl acetate, linalool, chamazulene and β-pinene, have been identified in plant oil across Europe [31,32,33,34,35,36]. However, our sample was devoid of *cis*-epoxycimene, chrysanthenol, chrysanthenyl acetate and β pinene. Another interesting compound identified in our sample was arborescin, a sesquiterpene compound scarcely found in wormwood oil from other geographical regions. It is worth noting that sage and lavender essential oils, whose main components include, as in our oil, camphor, α-thujone, and terpinen-4-ol, showed promising antioxidant, anti-inflammatory, and antiviral activity, making these oils and compounds good candidates for the development of new antiviral agents [37].

**Figure 1:**
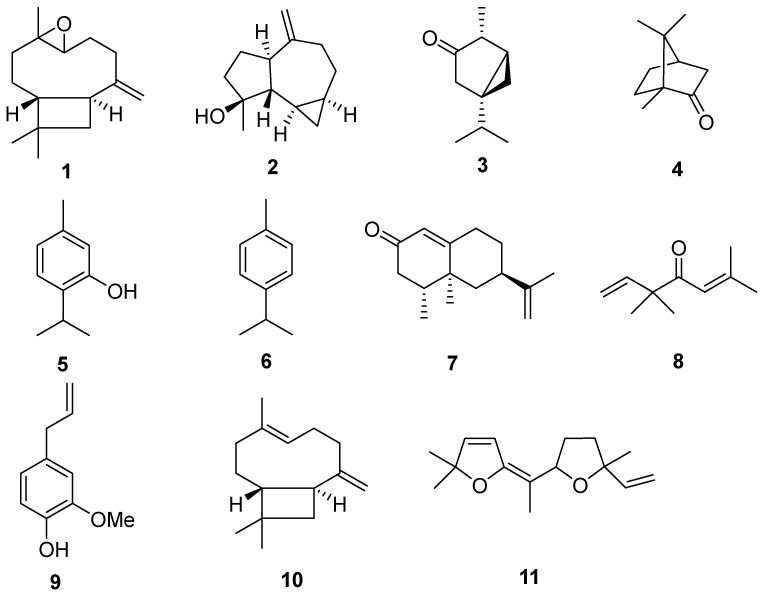
Major compounds identified in the essential oils of six plants: caryophyllene oxide (**1**), spathulenol (**2**), α-thujone (**3**), camphor (**4**), thymol (**5**), p-cymene (**6**), longiverbenone (**7**), artemisia ketone (**8**), eugenol (**9**), *trans*-caryophyllene (**10**) and davana ether (**11**).

The chemical composition of *E. globulus labill* comprised 67 compounds, representing 99.26% of the total oil (Table 2). Our oil analysis detected spathulenol (**2**) (structure shown in Figure 1) as an abundant component at 15%, whereas the predominant compounds, 1,8-cineole or eucalyptol, the best-known molecules in eucalyptus oil, accounted for only 4.52%. These results contradict those of another Moroccan study conducted in the province of Ouejda, where the results revealed 79.85% 1,8-cineole, whereas no trace of spathulenol was detected (**2**) [38]. On the other hand, other molecules were determined in our study to be major compounds but were absent in other works [39,40,41], such as caryophyllene oxide (**1**) (7.67%), *trans*-caryophyllene (**10**) (7.33%) and farnesol (7.52%). 1,8-Cineole, major component of this essential oil, is known for its antiviral potential against different human pathogen DNA and RNA viruses including rhinoviruses. Additionally, a recent permit proposes that this substance, the major propones que esta sustancia, componente mayor component of Coldmix^®^—a commercially available *Eucalyptus aetheroleum* and *Abies aetheroleum* blend for medicinal applications (Anadolu Hayat Essence & Chemical Ind. Co., Eskişehir, Turkey), may contribute or is even responsible for the anticovid activity attributed to this mixture [42].

**Figure 2:**
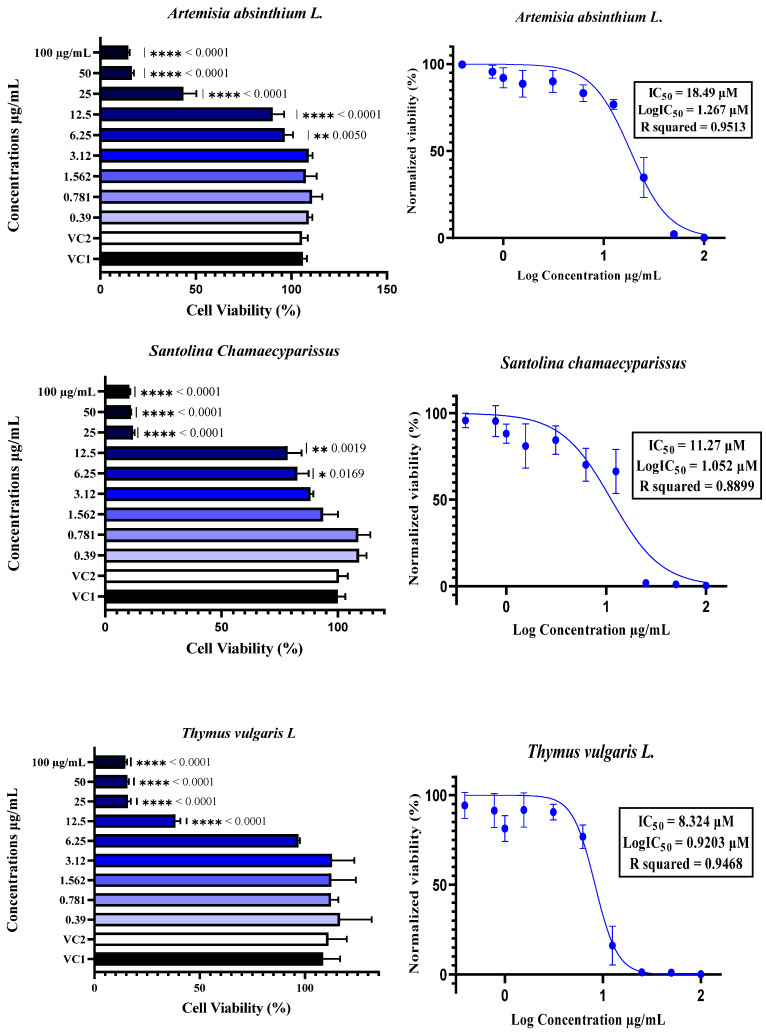

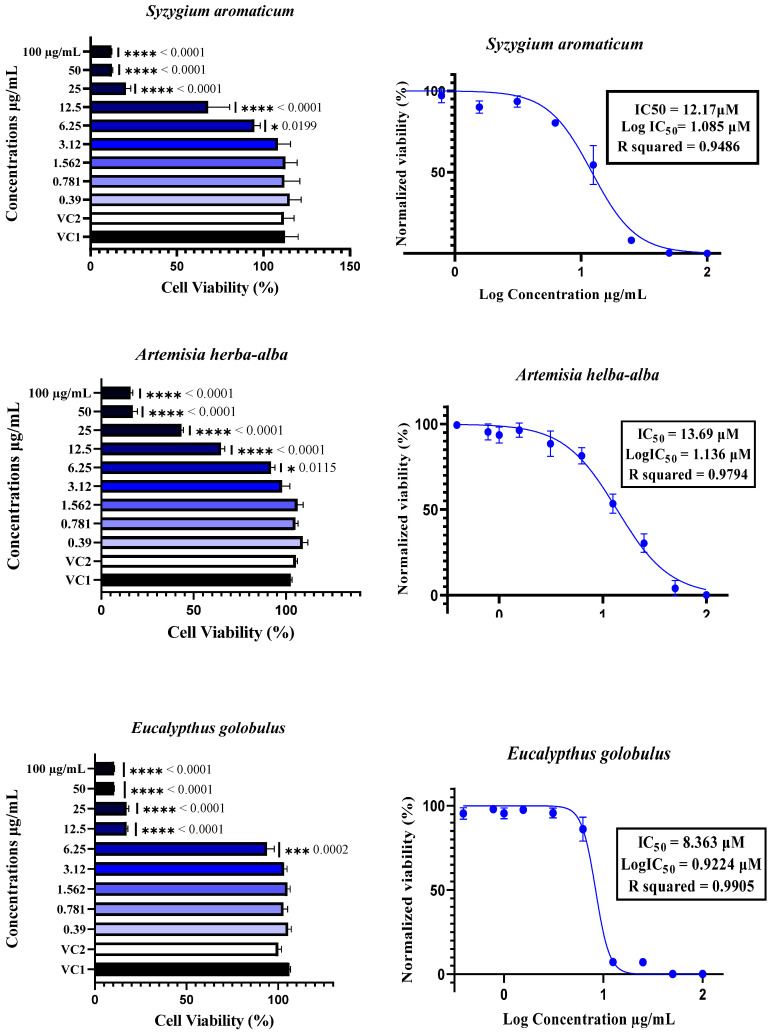
The cytotoxicity of plant essential oils (EOs) to Vero E6 cells was assessed by treating the cells with EOs at concentrations ranging from 0.39 µM to 100 µM. Cytotoxicity was determined using the neutral red uptake assay, with the results expressed as a percentage of cell viability compared with Vehicle Control 1 (VC1: untreated cells) and Vehicle Control 2 (VC2: DMSO/TWEEN 20-treated cells). The data are expressed as the means ± SDs, *n* = 4. The half-maximal inhibitory concentration (IC_50_) was calculated by normalizing the data and applying nonlinear regression analysis. Comparisons between different concentrations and controls were performed using one-way ANOVA followed by Dunnett’s multiple comparisons test, with statistical significance set at *p* < 0.05. In the figures, * indicates *p* < 0.05, ** indicates *p* < 0.01, *** indicates *p* < 0.001, and **** indicates *p* < 0.0001.

The chromatographic profile of *S. aromaticum oil* (Figure 2) revealed 16 compounds, representing 99.72% of the total oil content (Table 3). The main compound was eugenol (**9**) (54.96%), followed by *trans*-caryophyllene (**10**) (29.18%). These molecules are the best-known clove essential oils in various studies [13,14,15,16]. In addition to these two molecules, two components were interestingly found in our sample, α-cubebene (1.34%) and α-copaene (1.9%), which are absent from most of the publications cited above. Significantly, in silico studies indicate that eugenol exhibits a strong affinity for the structural components of SARS-CoV-2. Additionally, the oral administration of eugenol proved to reduced COVID-19 symptoms in mice treated with the SARS-CoV-2 spike S1 protein [43].

The characterization of *T. vulgaris* essential oil via GC–MS analysis revealed a total of 40 compounds, collectively accounting for 100% of the composition of the oil. Among these, 14 compounds were found to have concentrations greater than 1%, with individual percentages ranging from 1.28% to 33.33% (Table 4). The major component identified was thymol (**5**), an active monoterpene, which comprised 33.33% of the essential oil. This was followed by significant quantities of its terpenic hydrocarbon precursors, *p*-cymene (**6**) (25.87%) and γ-terpinene (7.21%). Additionally, a noteworthy concentration of carvacrol (5.23%), which is another phenolic monoterpene related to thymol (**5**), was observed. Furthermore, camphor was demonstrated to be a chemical type in thyme essential oil from eastern Morocco at a concentration of 39.39%, but was not detected in our study [44]. With regard to the antiviral activity of thymol, in vitro studies revealed its antiviral potential against SARS-CoV-2, probably due to its phenol ring structure [45].

For *A. herba-alba*, 50 compounds were detected in the GC/MS analysis, representing 96.73% of the total oil (Table 5). Among these compounds, davana ether (**11**) was found to be the main compound, with a concentration of 14.48%. Notably, we did not detect the presence of camphor in our analysis. Camphor is a compound whose content is particularly high in *A. alba* [44,46,47]. In another Moroccan study, davanone was identified as the main chemotypic compound of the essential oil, followed by davana ether (**11**) [48]. This suggests the presence of specific and advanced biosynthetic pathways. Other davanone derivatives, such as nordavanone (2.19%) and davana furan (1.31%), have also been detected; these compounds are valuable ingredients in perfumery and aromatherapy and are generally isolated from the essential oil of *Artemisia pallens* [48]. Three other oxygenated monoterpenes were recognized as fingerprints of this essential oil in addition to those mentioned above: chrysanthenone, *cis* chrysanthenyl acetate and 1,8-cineole, as published in [49,50], where they were classified as major compounds compared with the low traces found in our sample (*cis* chrysanthenyl acetate (0.97%) and 1,8-cineole (0.67%)) (see the full Table S5). This may be explained by the difference in harvesting time, since camphor plays a role in the chrysanthenone biosynthesis pathway.

*S. chamaecyparissus* essential oil was also studied, yielding 70 compounds, accounting for 99.98% of the oil (Table 6). The predominant compound observed was longiverbenone (**7**) (nootkatone), with a proportion of 18.15%, followed by artemisia ketone (**8**) (15.58%). This contrasts with previous reports [23,51], in which artemisia ketone (**8**) was detected as the main compound, with a proportion close to 15.65%, whereas nookatone (**7**) accounted for only 6.97%. According to numerous revised articles, various chemotypes from different regions have been noted to be completely devoid of nootkatone (**7**) [52,53,54,55,56,57]. Other compounds known as fingerprints of this plant were detected in different proportions, such as α-curcumene (4.82%), spathulenol (**2**) (4.41%) and epizanone (3.19%). It is worth noting that nootkatone, the major component of this oil proved to moderately inhibit SARS-CoV-2 [58]. A complete list of all the identified compounds and their characteristics can be found in the Supplementary Materials.

### 2.2. Cytotoxicity of Essential Oils

**Figure 3:**
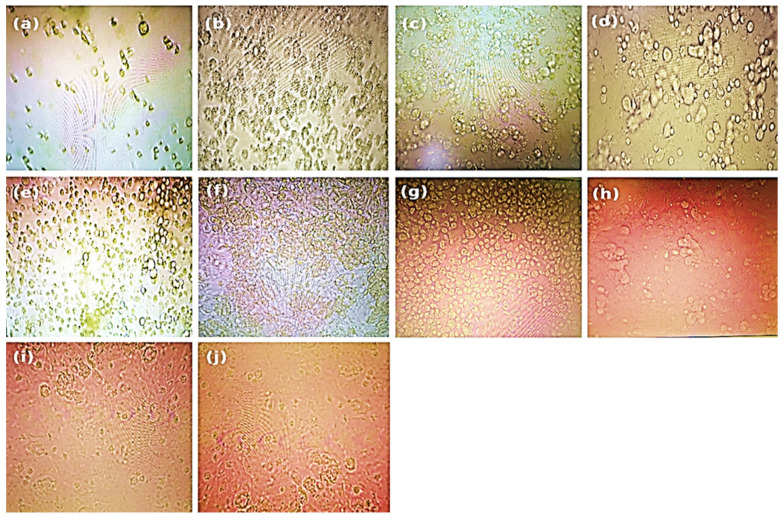
Microscopic evaluation of the monolayers revealed dose-dependent cytotoxic effects, with particular attention to cell rounding and other prominent morphological alterations compared with those of the control cells after 48 ± 2 h of incubation with the treatment concentrations, prior to performing the neutral red assay. The treatments used were as follows: (**a**) 100 µg/mL, (**b**) 50 µg/mL, (**c**) 25 µg/mL, (**d**) 12.5 µg/mL, (**e**) 6.25 µg/mL, (**f**) 3.12 µg/mL, (**g**) 1.562 µg/mL, (**h**) 0.781 µg/mL, (**i**) 0.39 µg/mL, and (**j**) untreated control.

The cytotoxicity of essential oils was evaluated in Vero E6 cells, a widely accepted model for SARS-CoV-2 research, using a neutral red uptake assay after 48 h of incubation at concentrations ranging from 0.39 to 100 µg/mL (Figure 3). The results revealed a clear dose-dependent cytotoxic effect, with essential oils exhibiting significantly reduced cell viability at concentrations ≥12.5 µg/mL, as confirmed by one-way ANOVA (*p* < 0.05) (Figure 2). The IC_50_ values varied notably among the six essential oils, ranging from 8.32 µM for *T. vulgaris* to 18.49 µM for *A. absinthium*. This variation appears to be strongly linked to the chemical composition of the oils. *T. vulgaris*, which had the greatest degree of cytotoxicity, contains thymol (**5**) and carvacrol, both of which are phenolic compounds with well-documented membrane-disruptive and apoptosis-inducing effects on cancer and epithelial cell lines [59,60,61,62]. However, the relatively lower concentration of carvacrol (5.23%) in our sample than the 25.5% reported in [16] could explain the moderate toxicity compared with the previously reported CC_50_ value of 2 µg/mL. *E. globulus* oil, which had similar cytotoxic potency (IC_50_ = 8.36 µM), had a different chemical profile than typically reported: instead of a high content of 1,8-cineole (86.6%) [16], our sample was rich in spathulenol (**2**) (15%), an alcohol with known moderate cytotoxic properties [16,63,64]. *S. aromaticum* presented an IC_50_ of 12.17 µM, which may be attributed to its eugenol (**9**) content (54.96%), a phenolic compound associated with strong cytotoxic effects [59,65]. Similarly, *S. chamaecyparissus* displayed an IC_50_ of 11.27 µM, which could be explained by the unusually high content of nootkatone (**7**) (18.15%) in our sample. Nootkatone (**7**), a sesquiterpenoid ketone rarely identified in Santolina oils, has shown potent cytotoxic activity in HL-60 and retinoblastoma cell lines, which is attributed to ROS generation, NF-κB suppression, autophagy induction, and cell cycle arrest [66,67,68,69]. In the case of *A. herba-alba* (IC_50_ = 13.69 µM) and *A. absinthium* (IC_50_ = 18.49 µM), the variability in toxicity relative to the literature data [70,71,72] can likely be attributed to differences in the chemotype and constituent ratios. The greater toxicity of *A. absinthium* in our study may be explained by its significant thujone (**3**) content (29.02%), a monoterpene shown to be cytotoxic at low micromolar concentrations across multiple cancer cell lines [73,74]. These findings highlight that its cytotoxicity is not solely dependent on major constituents but also on the complex interactions among minor compounds, which may modulate their absorption, solubility, and bioavailability [63,75]. Additionally, functional group analysis from previous chemometric studies [16] supports our observations, showing that phenols, alcohols, aldehydes, and esters tend to increase cytotoxic and antiviral activity, whereas ethers and hydrocarbons may reduce it. Therefore, the stronger cytotoxic effects observed in *T. vulgaris*, *S. aromaticum*, and *E. globulus* may be attributed directly to the relative abundance of active functional groups. Overall, the observed cytotoxic profiles are strongly correlated with the phytochemical composition, the concentration and type of functional groups present, and the potential synergistic interactions between constituents, confirming the relevance of the essential oil chemotype in determining bioactivity.

### 2.3. Antimicrobial Activity

The antimicrobial efficacy of the essential oils, as presented in Table 7, was evaluated against standard antibiotics and antifungal agents (Table 8) using inhibition zone diameters as the primary metric. *E. globulus* showed moderate activity against *E. coli* (11 mm; +), although it was less effective than gentamicin and nalidixic acid (18 mm; ++), which is consistent with prior reports indicating limited gram-negative activity for *Eucalyptus* species, with minimum inhibitory concentrations (MICs) ranging from 1000–2000 µg/mL [17]. This moderate efficacy may reflect the specific phytochemical profile of the tested oil, which included compounds such as farnesol, which was shown to exhibit antimicrobial activity against *E. coli* and *Aspergillus niger*, with inhibition zones of 12 mm and 11 mm, respectively, at 50 μg/mL [76]. *P. aeruginosa* was resistant to all the essential oils tested and showed only minimal sensitivity to gentamicin (10 mm; +), reflecting its well-known resistance mechanisms, including a restrictive outer membrane [77] and active efflux pumps [78] that limit the intracellular accumulation of lipophilic agents such as terpenes and phenolics.

In contrast, gram-positive bacteria such as *B. subtilis* were more responsive: *E. globulus* exhibited extreme sensitivity (+++, 20 mm), comparable to nalidixic acid. This heightened activity may be linked to better membrane permeability and the presence of bioactive components such as 1,8-cineole, spathulenol, β-caryophyllene, and caryophyllene oxide, all of which have demonstrated antimicrobial activity depending on their concentration and synergistic interactions [79,80]. *S. aromaticum* and *A. herba-alba* also showed moderate sensitivity (++) to *B. subtilis*, likely due to the high eugenol content in clove oil, which results in strong antimicrobial effects across a wide spectrum of gram-positive and gram-negative bacteria as well as fungi [81]. *A. herba-alba* contains potent phenolic and ketonic components that have demonstrated high antimicrobial activity against *S. aureus* and *Shigella sp*. at low concentrations (0.07–10 mg/mL) [82].

Similarly, *T. vulgaris* exhibited moderate inhibitory effects (15 mm; +) on *S. aureus*, which is consistent with the high levels of thymol and carvacrol. These phenolic compounds target bacterial membranes, disrupt metabolic processes, and are also effective against multidrug-resistant strains such as nontuberculous mycobacteria, with MICs between 32–128 µg/mL [83]. In contrast, *S. epidermidis* exhibited limited sensitivity to essential oils, whereas gentamicin showed moderate efficacy (12 mm; +). This reduced susceptibility may be due to biofilm formation and gene transfer mechanisms that increase resistance [7].

For *C. albicans*, *S. chamaecyparissus* showed slight sensitivity (+, 11.75 ± 0.1 mm), whereas itraconazole exhibited extreme sensitivity (+++, 19 mm). This reduced antifungal effect may stem from differences in essential oil composition, including lower levels of potent antifungals such as nootkatone or a greater proportion of less active monoterpenes [69]. These findings contrast with those of Abd El-Baky et al. [84], who reported strong antifungal activity in oils rich in borneol, linalool, and eugenol. Additionally, *Candida glabrata* ATCC 28226 and *Malassezia furfur* ATCC 4342 were tested but showed no sensitivity to any of the essential oils evaluated, indicating that further investigations are needed to better understand these fungal responses. While the antifungal data are limited, the results against *C. albicans* suggest potential for *S. chamaecyparissus*, which warrants further exploration.

Importantly, the antimicrobial performance of these essential oils closely correlated with their dominant phytochemical profiles. The presence of phenolic compounds (eugenol, thymol, carvacrol), ketones (thujone, artemisia ketone, nootkatone), and sesquiterpenes (spathulenol, caryophyllene oxide) was consistently associated with stronger antimicrobial effects. These compounds act through various mechanisms, including disruption of cell membranes, inhibition of metabolic enzymes, and interference with quorum sensing. This structure–activity relationship underscores the need for detailed chemical profiling when evaluating essential oil bioactivity and supports their potential role, although currently limited, as adjuncts in antimicrobial treatment strategies.

The obtained results show poor agreement with previously reported data. The differences observed between our findings and those in the literature can be attributed to a combination of ecological, genetic, and methodological factors known to influence essential oil biosynthesis. The qualitative and quantitative composition of essential oils is strongly affected by environmental parameters such as altitude, temperature, humidity, photoperiod, and soil nutrient availability, all of which modulate the enzymatic pathways involved in terpene formation [1,85].

In the case of *A. absinthium*, the predominance of α-thujone and camphor observed in Moroccan samples may be linked to enhanced activity of the sabinene-derived thujone biosynthetic pathway, which has been characterized in related *Artemisia* species [86]. Environmental stressors typical of semi-arid regions, such as elevated temperature and solar radiation, are known to influence monoterpene metabolism and could thereby favor α-thujone accumulation [87]. The comparatively low β-thujone content may reflect population-specific chemotypic variation rather than differences in isomerization efficiency [88].

Similarly, in *Eucalyptus globulus*, the relatively low proportion of 1,8-cineole and the higher abundance of spathulenol may reflect species- and environment-specific variability in volatile composition. In *Eucalyptus*, temperature and other abiotic factors, which are typical of Moroccan coastal ecosystems, are known to influence monoterpene emissions and alter the relative abundance of compounds, while variation in essential oil composition across taxa and populations is largely determined by species identity, developmental stage, and growth conditions [89,90]. Additionally, Differences may also arise from post-harvest and methodological factors. The moisture level and storage duration of plant material prior to hydrodistillation can alter volatile compound stability, while the distillation time, temperature, and apparatus configuration can influence the relative recovery of low-boiling versus high-boiling constituents [91]. In *Thymus vulgaris*, the observed predominance of thymol over its precursors p-cymene and γ-terpinene likely reflects the harvest stage, since oxidative conversion of γ-terpinene to thymol increases near flowering [92]. Chemotypic variation is further enhanced by genetic polymorphism and potential hybridization within plant populations, giving rise to locally adapted chemical phenotypes [93].

Overall, the enrichment of oxygenated terpenes (e.g., spathulenol, camphor, thymol) in our samples may enhance antimicrobial potency and cytotoxic selectivity, suggesting that Moroccan chemotypes could represent valuable sources of bioactive compounds for pharmaceutical and therapeutic applications.

## 3. Materials and Methods

### 3.1. Plant Collection

Aromatic medicinal herbs (*Artemisia absinthium* L., *Syzygium aromaticum, Artemisia herba-alba*, and *Eucalyptus globulus*) were procured from a local herb store in Rabat, Morocco, in March 2022. The medicinal plants were identified morphologically with the assistance of Professor Khamar Hamid from the Botany Department at the Scientific Institute of Mohammed V University in Rabat, Morocco. Additional plant species, including *Santolina chamaecyparissus* and *Thymus vulgaris* L., were provided in May 2022 by the National Agency for Medicinal and Aromatic Plants (ANPMA) in Taounate, Morocco. This study utilized the aerial parts of *Artemisia absinthium* L., *Santolina chamaecyparissus*, *Artemisia herba-alba*, *Eucalyptus globulus*, and *Thymus vulgaris* L., as well as the seeds *of Syzygium aromaticum*, to accurately reflect their traditional usage by the Moroccan population.

### 3.2. Extraction of Essential Oils

The fresh aerial parts were dried at room temperature in the dark in an open drying room for ten days. After drying, they were pulverized into small pieces using a Willy mill and labeled. The essential oils from the six species were prepared using a conventional hydrodistillation method in a Clevenger-type apparatus, following a slightly modified version of the method described by [94]. Specifically, 200 g of each sample was accurately weighed and combined with 1 L of distilled water in a 2 L flask connected to a Clevenger-type apparatus with a Dean–Stark distillation tank. The mixture was heated to 100 °C for 3 h, as per the standard procedure of the European Pharmacopoeia. The concentrated essential oil was then manually separated from the aromatic water and dried over anhydrous sodium sulfate to remove any residual moisture, ensuring that the essential oil remained dry. The final extracts were stored in amber-colored bottles at −20 °C for subsequent cytotoxicity testing.

### 3.3. Gas Chromatography–Mass Spectroscopy Analysis

The essential oils were analyzed using gas chromatography–mass spectrometry (GC–MS) by a Shimadzu GC-2010 gas chromatograph coupled to a Shimadzu GCMS-QP2010 Ultra mass detector (Shimadzu Corporation, Kyoto, Japan) operating with electron ionization at 70 eV. Sample injections (1 μL) were performed using an AOC-20i autosampler, and a 30 m × 0.25 mm i.d. capillary column with a 0.25-μm film thickness Teknokroma (Barcelona, Spain) TRB-5 (95% dimethyl-diphenylpolysiloxane, 5%) was used. The operating conditions were as follows: split ratio of 20:1, injector temperature of 300 °C, transfer line temperature of 250 °C, and initial column temperature of 70 °C. The temperature was then increased to 290 °C at a rate of 6 °C/min. Full-scan mode (*m*/*z* 35–450) was used for detection. The identities of the compounds were determined by comparing their electron ionization mass spectra and retention data with those in the Wiley 229 and NIST 17 mass spectral databases. For quantification, the relative area percentages of all peaks obtained in the chromatograms were used. All extracts (4 μg/μL) were dissolved in 100% dichloromethane for injection.

### 3.4. Cytotoxicity Assay of Essential Oils

#### 3.4.1. Vero-E6 Cells

The potential effects of essential oils on cell morphology and viability were investigated in vitro using the Vero-E6 cell line, which has been shown to be highly sensitive to the SARS-CoV-2 virus. The cell line used in this study corresponds to green monkey kidney fibroblasts (Cercopithecus aethiops) maintained in continuous culture (ATCC-CRL-1586, Vero cells, passage 3). The cells were originally obtained from the American Type Culture Collection (ATCC, Manassas, VA, USA) and provided for this study by the Centre National de Référence (CNR), Lyon, France.

#### 3.4.2. Culture Medium and Preparation of Plates

The culture medium used for maintaining and growing the cells was Dulbecco’s modified Eagle’s medium (DMEM) (Gibco, Vacaville, CA, USA), which was modified with Earle’s salts (Sigma Aldrich, St. Louis, MO, USA) and nonessential amino acids. The medium was buffered with 7.5% sodium bicarbonate to a pH of 6.90 ± 0.1 under CO_2_. Sterile fetal bovine serum (FBS) (10%) (Gibco, USA) was added to the DMEM. Vero-E6 cells were cultured as monolayers in tissue culture flasks (75–25 cm^2^) under standard conditions of 37 °C ± 1 °C, 90% ± 5% humidity, and 5.0% ± 1% CO_2_/air. The cell morphology was monitored daily using a phase-contrast microscope to ensure optimal growth conditions. After reaching 80% confluence, the cells were detached from the flasks with 0.5% trypsin diluted in PBS (Sigma Aldrich, St. Louis, MO, USA). For the experiment, a 3.105 cells/mL cell suspension was prepared and added to 96-well microtiter plates (200 µL per well). The plates were then incubated for 24 ± 2 h under the same conditions to allow for cell recovery and adhesion, forming a monolayer of less than 50% confluence.

#### 3.4.3. Preparation of Essential Oils

To prepare the essential oils, 10 µL of each oil (equivalent to 1 mg) was solubilized in a mixture of two emulsifiers, DMSO and TWEEN 20, at a concentration of 2% (*v*/*v*). This mixture was optimized to ensure complete solubilization and improve the diffusion of volatile molecules while minimizing toxicity to the cells. The resulting homogeneous stock solution for each essential oil was then prepared for the assay. Each stock solution was further diluted in culture medium (without FBS) using a titration plate. The initial dilution was 1/10, followed by a series of double dilutions over a range of concentrations from 100 µg/mL to 0.39 µg/mL.

#### 3.4.4. Cytotoxicity Evaluation via the Neutral Red Colorimetric Assay (NR)

The cytotoxicity of the selected essential oils was evaluated at the National Hygiene Institute in Rabat, Morocco, using the neutral red uptake (NRU) assay, which is based on the method described in [95] with some optimizations. The NRU assay uses neutral red dye, a weak cationic dye that readily penetrates cell membranes by passive nonionic diffusion. The dye accumulates in lysosomes, where it binds to anionic and phosphate groups in the lysosomal matrix via electrostatic and hydrophobic interactions.

The essential oils were tested at various concentrations, ranging from 100 to 0.39 µg/mL, in quadruplicate for each. Two control columns were included: one containing the respective solvent and the other containing untreated cells. Immediately after the oils were added, adhesives were applied to the plates to prevent contamination by vapors from high doses. The plates were incubated for 48 h at 37 °C in a 5% CO_2_ atmosphere. After the incubation period, the cells were rinsed with prewarmed D-PBS to remove any residual oil. The cells were then incubated for 3 h in neutral red medium prepared in DMEM at a concentration of 40 µg/mL to allow for the formation of NR crystals, indicating cell viability. Following the 3-h incubation, the NR medium was removed, and the cells were rinsed with D-PBS before adding a NR Desorb solution consisting of 50% ethanol, 96%, 49% deionized water, and 1% glacial acetic acid to extract the NR dye from the cells. The plates were shaken for 10 min to ensure thorough extraction, and absorbance measurements were then taken at 540 nm ± 10 nm using a microplate reader (Multiskan EX, Thermo Scientific).

Cell viability was calculated using the following formula:

% Cell viability = (OD of essential oils/OD of control cells) ×100

where OD refers to optical density, control cells refer to solvent-treated cells, and the OD of essential oils refers to treated cells.

### 3.5. Antimicrobial Activity

#### 3.5.1. Microbial Strains

The eight microbial strains used in this study were selected as standard reference strains commonly employed for evaluating antimicrobial and antifungal activity. These include two gram-negative bacterial strains: *Escherichia coli* ATCC 25922 and *Pseudomonas aeruginosa* ATCC 27853; three gram-positive strains: *Staphylococcus epidermidis* ATCC 12228, *Bacillus subtilis* ATCC 6633, and *Staphylococcus aureus* ATCC 25923; and three fungal strains: *Candida albicans* ATCC 10231, *Candida glabrata* ATCC 28226, and *Malassezia furfur* ATCC 4342.

#### 3.5.2. Preparation of the Inoculums

Before antimicrobial tests were conducted, all microbial strains were subcultured under appropriate conditions to ensure viability and standardized growth. Bacterial strains (*E. coli*, *P. aeruginosa*, *S. epidermidis*, *B. subtilis*, and *S. aureus*) were incubated overnight at 37 °C for 24 h on Mueller–Hinton agar (MHA). Fungal strains (*C. albicans*, *C. glabrata*, and *M. furfur*) were cultured on potato dextrose agar (PDA) and incubated at 30 °C for 48 h. Well-isolated colonies were collected using a sterile platinum loop and suspended in sterile saline solution. The optical density of the bacterial suspensions was adjusted to a range of 0.4–0.6 at 405 nm using a spectrophotometer, corresponding to a final concentration of 10^6^–10^7^ CFU/mL [96].

#### 3.5.3. Disk Diffusion Method on Agar

To evaluate the sensitivity of the microbial strains to the different essential oils, a disk diffusion method was employed [96]. Briefly, 10 µL of diluted essential oil samples (20 mg/mL, dissolved in 10% DMSO/0.5% Tween 20) were applied to sterile filter paper discs with a diameter of 6 mm. The discs were then placed on Mueller–Hinton agar previously inoculated with the test microorganisms using a sterile loop. For the fungal strains, potato dextrose agar was used. A disk with an equivalent volume of 10% DMSO/0.5% Tween 20 served as the negative control. The positive controls included commercially available antibiotic discs (Bio-Rad, Hercules, CA, USA), such as gentamicin (10 μg), nalidixic acid (30 μg), ampicillin (10 μg), spectinomycin (100 μg), penicillin (6 μg), fluconazole (30 μg), and itraconazole (30 μg). The Petri dishes were incubated at 4 °C for 2 h to facilitate compound diffusion. The diameters of the inhibition zones were subsequently measured in millimeters after 24 h of incubation at 37 °C for bacteria and 48 h at 28 °C for yeast.

The results are expressed as the diameter of the inhibition zone and are categorized according to the sensitivity of the strains to the extracts as follows [97]:

- Not Sensitive (−) or Resistant: Diameter < 8 mm
- Sensitive (+): Diameter between 9 and 14 mm
- Very Sensitive (++): Diameter between 15 and 19 mm
- Extremely Sensitive (+++): Diameter > 20 mm

### 3.6. Data Analysis

Relative cell viability was expressed as a percentage of the vehicle control (VC). The mean neutral red uptake (NRU) values from four replicates per concentration were calculated, with blank values subtracted. The IC50 value was determined by fitting a nonlinear regression curve to the normalized data using GraphPad Prism software version 9.1.0, with the results reported as the means ± SDs Statistical differences between the experimental and control values were analyzed using one-way ANOVA followed by Dunnett’s multiple comparisons test, with the significance level set at *p* < 0.05.

## 4. Conclusions

In summary, this study evaluated the cytotoxic effects of various essential oils extracted from aromatic medicinal plants commonly used in Morocco for COVID-19 prevention and treatment via Vero E6 cells. *T. vulgaris* L., *E. globulus*, and *S. chamaecyparissus* presented the most potent cytotoxic activities. The variations in cytotoxicity compared with those reported in previous studies were attributed to differences in chemical composition, cell lines, and assay methodologies. Key compounds such as thymol, carvacrol, eugenol, and thujone significantly contributed to the observed effects.

The assessment of antimicrobial activity against a range of standard bacterial and fungal strains revealed variable effectiveness. Differences in strain biology and essential oil chemistry were highlighted, demonstrating the limited potential of these oils as alternatives to traditional treatments for resistant strains. Further investigation is necessary to understand the mechanisms of action of essential oils and their active compounds and to optimize their use in antimicrobial therapies.

These findings support the need for continued research to explore the therapeutic potential of these essential oils in treating COVID-19 and other viral infections. Future studies should include comprehensive in vitro and in vivo analyses to better understand the antiviral mechanisms and validate the efficacy of these oils against SARS-CoV-2. Our laboratory is currently investigating the antiviral properties of these essential oils, which may lead to novel, plant-based therapeutic strategies for combating viral infections.

## Figures and Tables

**Table 1:** Chemical composition of *A. absinthium* L. (boldface indicates a major compound).

No.	Compound ^a^	Ret. Time (min)	Relative Content (%)	Molecular Formula
**1**	Sabinene	5.10	1.64	C_10_H_16_
**2**	β-Myrcene	5.29	2.58	C_10_H_16_
**3**	γ-Terpinene	6.74	1.32	C_10_H_16_
**4**	*trans* Sabinene hydrate	7.03	1.54	C_10_H_18_O
**5**	Linalool	7.56	1.06	C_10_H_18_O
**6**	β-Thujone	7.88	1.67	C_10_H_16_O
**7**	**α-Thujone**	8.15	**29.02**	C_10_H_16_O
**8**	**Camphor**	8.92	**24.34**	C_10_H_16_O
**9**	**(−)-4-Terpineol**	9.57	**3.68**	C_10_H_18_O
**10**	Caryophyllene	15.14	1.23	C_15_H_24_
**11**	Germacrene-D	16.50	2.65	C_15_H_24_
**12**	3,6-Dihydrochamazulene	17.11	1.52	C_14_H_16_
**13**	Elemol	17.89	1.53	C_15_H_26_O
**14**	**Chamazulene**	21.77	**6.92**	C_14_H_16_
**15**	Arborescin	26.18	2.18	C_15_H_24_
**16**	*geranyl*-α-terpinene	26.49	2.14	C_15_H_24_

^a^ Molecules higher than 1%.

**Table 2:** Chemical composition of *E. globulus* (boldface indicates a major compound).

No.	Compound ^a^	Retention Time (min)	Relative Content (%)	Molecular Formula
**1**	α-Pinene	4.47	3.6	C_10_H_16_
**2**	***p*-Cymene**	6.08	**6.29**	C_10_H_14_
**3**	Limonene	6.16	1.26	C_10_H_16_
**4**	β-Phellandrene	6.24	4.2	C_10_H_16_
**5**	**1,8-Cineole**	6.27	**4.52**	**C_10_H_18_O**
**6**	Linalool	7.56	1.02	C_10_H_18_O
**7**	4-Terpineol	9.58	2.33	C_10_H_18_O
**8**	Crypton	9.81	1.82	C_10_H_16_O
**9**	α-Terpineol	9.90	3.95	C_10_H_18_O
**10**	Copaene	14.08	1.08	C_15_H_24_
**11**	***trans*-Caryophyllene**	15.15	**7.33**	**C_15_H_24_**
**12**	Neolloocimene	16.05	2.39	C_15_H_24_
**13**	**Spathulenol**	18.62	**15**	**C_15_H_24_O**
**14**	**Caryophyllene oxide**	18.77	**7.67**	**C_15_H_24_O**
**15**	Viridiflorol	19.01	2.23	C_15_H_26_O
**16**	Isoleptospermone	19.16	1.14	C_15_H_22_O_3_
**17**	*cis*-Eudesm-6-en-11-ol	19.22	1.08	C_15_H_26_O
**18**	Aromadendrane-4,10-diol	19.72	1.59	C_15_H_26_O_2_
**19**	**Farnesol**	21.17	**7.52**	**C_15_H_26_O**
**20**	Valerenal	21.79	1.18	C_15_H_22_O
**21**	(1S,4R,5S)-1-Methyl-4- (prop-1-en-2-yl)spiro [4.5] dec-7-ene-8-carbaldehyde	22.18	2.15	C_15_H_24_O

^a^ Molecules higher than 1%.

**Table 3:** Chemical composition of *S. aromaticum* (boldface indicates a major compound).

No.	Compound ^a^	Retention Time (min)	Relative Content (%)	Molecular Formula
**1**	α-Cubebene	13.36	1.34	C_15_H_24_
**2**	**Eugenol**	**13.60**	54.96	C_10_H_12_O_2_
**3**	α-Copaene	14.09	1.9	C_15_H_24_
**4**	***trans*-Caryophyllene**	**15.17**	29.18	C_15_H_24_
**5**	α-Humulene	15.96	3.79	C_15_H_24_
**6**	Eugenol acetate	17.11	5.41	C_12_H_14_O_3_

^a^ Molecules higher than 1%.

**Table 4:** Chemical composition of *T. vulgaris* (boldface indicates a major compound).

No.	Compound ^a^	Ret. Time (min)	Relative Content (%)	Molecular Formula
**1**	Amyl vinyl carbinol	5.12	1.78	C_7_H_14_O
**2**	β-Myrcene	5.29	1.82	C_10_H_16_
**3**	α-Terpinene	5.92	1.28	C_10_H_16_
**4**	***p*-Cymene**	**6.09**	**25.87**	C_10_H_14_
**5**	1,8-Cineole	6.27	2.39	C_10_H_18_O
**6**	**γ-Terpinene**	**6.74**	**7.21**	C_10_H_16_
**7**	Linalool	7.56	3.34	C_10_H_18_O
**8**	L-Borneol	9.43	2.26	C_10_H_18_O
**9**	4-Terpineol	9.58	2.03	C_10_H_18_O
**10**	Isothymol methyl ether	10.81	1.41	C_11_H_16_O
**11**	**Thymol**	**12.05**	**33.33**	C_10_H_14_O
**12**	**Carvacrol**	**12.24**	**5.23**	**C_10_H_14_O**
**13**	Caryophyllene	15.15	1.44	C_15_H_24_
**14**	Caryophyllene oxide	18.76	2.19	C_15_H_24_O

^a^ Molecules higher than 1%.

**Table 5:** Chemical composition of *A. herba-alba* (boldface indicates a major compound).

No.	Compound ^a^	Ret. Time (min)	Relative Content (%)	Molecular Formula
**1**	Cyclohexanebutanal, 2-methyl-3-oxo-, *cis*-	4.65	1.41	C_11_H_18_O_2_
**2**	Decane, 2-cyclohexyl-	6.14	1.41	C_16_H_32_
**3**	*cis*-Arbusculone	6.59	1.17	C_15_H_24_O_2_
**4**	**β-Thujone**	**7.88**	**4.31**	**C_10_H_16_O**
**5**	**Thujone**	**8.13**	**4.09**	**C_10_H_16_O**
**6**	**(+)-2-Bornanone**	**8.90**	**6.02**	**C_10_H_16_O**
**7**	Nordavanone	10.53	2.19	C_10_H_14_O_2_
**8**	*Cis* jasmone	14.50	1.97	C_11_H_16_O
**9**	Davana furan	14.73	1.31	C_11_H_16_O
**10**	**2-Butyl-5-methyl-3-(2-methylprop-2-enyl) cyclohexanone**	15.88	2.49	C_14_H_24_O
**11**	**Davana ether**	**16.78**	**14.48**	**C_13_H_22_O_2_**
**12**	Artedouglasia oxide C	17.47	1.46	C_15_H_22_O_2_
**13**	1H-Cycloprop[e]azulen-7-ol, decahydro-1,1,7-trimethyl-4-methylene-, [1ar-(1a α, 4a α-,7 β, 7a β,7b α)]-	18.61	2.91	C_15_H_24_O
**14**	Caryophyllene oxide	18.75	1.36	C_15_H_24_O
**15**	**1-((1R,2R,3R)-2-(3-Isopropylfuran-2-yl)-3-methylcyclopentyl) ethanone**	18.94	1.5	C_13_H_20_O_2_
**16**	3-Methyl-but-2-enoic acid, 1,7,7-trimethyl-bicyclo [2.2.1] hept-2-yl ester	19.14	2.1	C_15_H_24_O_2_
**17**	Humulenol-II	19.56	1	C_15_H_26_O
**18**	**2-Propenal, 3-(2,6,6-trimethyl-1-cyclohexen-1-yl)-**	**20.29**	**5.58**	C_12_H_18_O
**19**	Davanone	20.65	3.21	C_14_H_22_O

^a^ Molecules higher than 1%.

**Table 6:** Chemical composition of *S. chamaecyparissus* (boldface indicates a major compound).

No.	Compound ^a^	Retention Time (min)	Relative Content (%)	Molecular Formula
**1**	β- Myrcene	5.29	1.28	C_10_H_16_
**2**	*P-C*ymene	6.08	1.3	C_10_H_14_
**3**	β-Phellandrene	6.23	2.73	C_10_H_16_
**4**	**Artemisia ketone**	**6.66**	**15.58**	C_10_H_16_O
**5**	*cis*-Chrysanthemyl alcohol	8.80	1.67	C_10_H_18_O
**6**	(+)-2-Bornanone	8.90	2.11	C_10_H_16_O
**7**	*endo*-Borneol	9.43	1.17	C_10_H_18_O
**8**	α-Longipinene	13.55	1.96	C_15_H_24_
**9**	γ-Curcumene	16.28	1.55	C_15_H_22_
**10**	**α-Curcumene**	**16.37**	**4.81**	**C_15_H_22_**
**11**	n-Pentadecanol	16.46	1.4	C_15_H_32_O
**12**	Germacrene-D	16.50	1.79	C_15_H_24_
**13**	δ-Cadinene	17.22	1.52	C_15_H_24_
**14**	Caryophyllene oxide	17.73	1.49	C_15_H_24_O
**15**	**Spathulenol**	**18.61**	**4.41**	**C_15_H_24_O**
**16**	Epizizanone	18.76	3.19	C_15_H_22_O
**17**	β-Oplopenone	19.15	1.46	C_15_H_22_O
**18**	(+)-*trans*-caran-*cis*-3-ol	19.21	1.82	C_10_H_18_O
**19**	(2R,3R,4aR,5S,8aS)-2-Hydroxy-4a,5-dimethyl-3-(prop-1-en-2-yl)-2,3,4,4a,5,6-hexahydronaphthalen-1(8aH)-one	19.99	2.77	C_15_H_24_O_2_
**20**	**Nootkatone**	**20.12**	**18.15**	C_15_H_22_O

^a^ Molecules higher than 1%.

**Table 7:** Comparative evaluation of essential oils for antimicrobial potential against reference microbial strain.

Essential Oils	Inhibition Zone (mm) for Reference Strains ^1^					
	Negative Gram (−)		Positive Gram (+)			Yeast Strain
	*Escherichia coli* (<0.0001)	*Pseudomonas aeruginosa*	*Bacillus subtilis* (<0.0001)	*Staphylococcus aureus* (<0.0001)	*Staphylococcus epidermidis* (=0.0010)	*Candida albicans* (<0.0001)
*E. globulus*	11.25 ± 0.6 ^b^	NA	20 ± 0.00 ^a^	NA	15 ± 0.7 ^a^	NA
*T. vulgaris* L.	NA	NA	NA	15.05 ± 0.7 ^b^	NA	NA
*A. absinthium*	NA	NA	NA	NA	NA	NA
*S. aromaticum*	NA	NA	12.75 ± 0.1 ^b^	NA	NA	NA
*A. helba-alba*	NA	NA	12.25 ± 0.1 ^b^	12.25 ± 0.1 ^c^	NA	NA
*S. chamaecyparissus*	NA	NA	10.00 ± 0.6 ^c^	NA	NA	11.75 ± 0.1 ^b^
Best Reference Antibiotic	18.25 ± 0.3 ^a^	10.25 ± 0.6	20.05 ± 0.00 ^a^	31.05 ± 0.4 ^a^	12.06 ± 0.4 ^b^	19 ± 0.00 ^a^

^1^ Diameter of the inhibition zone (mm), including a 6 mm disc, tested at a concentration of 0.2 mg/disc. NA indicates no activity. Values are the means ± SDs, *n* = 3. Statistical Analysis: One-way ANOVA (*p* < 0.001) followed by Tukey’s post-hoc test was applied when 3 or more treatments showed activity, a Welch’s *t*-test was used when exactly 2 treatments showed activity, no statistical analysis was performed when less than 2 treatments were active. Different superscript letters (e.g., a, b, c) indicate statistically significant differences between means at *p* < 0.05, with the order of letters reflecting the ranking of group means (e.g., a > b > c).

**Table 8:** Comparative antimicrobial efficacy of reference antibiotics and antifungal agents against reference microbial strain.

Microbial Strain	*Escherichia coli* (<0.0001)	*Pseudomonas aeruginosa*	*Bacillus subtilis* (<0.0001)	*Staphylococcus aureus* (<0.0001)	*Staphylococcus epidermidis* (=0.0004)	*Candida albicans*
Fluconazole 30 μg/mL	Nt	Nt	Nt	Nt	Nt	12 ± 0.00
Gentamicin 10 μg/mL	18.25 ± 0.3 ^a^	10.25 ± 0.6	NA	20 ± 0.1 ^b^	12.06 ± 0.4 ^a^	Nt
Nalidixic acid 30 μg/mL	18 ± 0.00 ^a^	NA	20.05 ± 0.00 ^a^	NA	10.25 ± 0.25 ^b^	Nt
Itraconazole 30 μg/mL	Nt	Nt	Nt	Nt	Nt	19 ± 0.00
Penicilin 6 μg/mL	10.62 ± 0.3 ^b^	NA	15.25 ± 0.6 ^b^	30.75 ± 0.2 ^a^	NA	Nt
Ampicillin 10 μg/mL	NA	NA	12.00 ± 0.00 ^c^	31.05 ± 0.4 ^a^	NA	Nt

NA indicates no activity and Nt not tested. Values are the means ± SDs, *n* = 3. Statistical Analysis: One-way ANOVA (*p* < 0.001) followed by Tukey’s post-hoc test. Different superscript letters (e.g., a, b, c) indicate statistically significant differences between means at *p* < 0.05, with the order of letters reflecting the ranking of group means (e.g., a > b > c).

## Data Availability

Data is contained within the article/Supplementary Materials.

## Supplementary Materials

The following supporting information can be downloaded at: https://www.mdpi.com/article/10.3390/molecules30214179/s1, Figure S1: Gas chromatography chromatograms of the essential oils extracted from the following plants: (**1**) *A. absinthium* L.; (**2**) *E. globulus*; (**3**) *T. vulgaris*; (**4**) *S. aromaticum*; (**5**) *A. herba-alba*; and (**6**) *S. chamaecyparisses*; Table: S1: Chemical composition of *Artemisia absinthium*; Table: S2: Chemical composition of *Eucalypthus globulus*; Table: S3: Chemical composition of *thymus vulgaris*; Table S4: Chemical composition of *Syzgium aromatic*; Table: S5: Chemical composition of *artemisia helba-alba*; Table: S6: Chemical composition of *Santolina chamaecyparisses*.

## Abbreviations

The following abbreviations are used in this manuscript:

EOs	Essential oils
GC-MS	Gas chromatography coupled to mass spectrometry
MIC	Minimal inhibitory concentration
IC_50_	Inhibitory concentration 50%
