# Supplementary material for: GC/MS Analysis, Cytotoxicity, and Antimicrobial Properties of Six Moroccan Essential Oils Traditionally Used for COVID-19 Prevention
Source: Molecules. 2025 Oct 25;30(21):4179. doi: 10.3390/molecules30214179 (PMC12610821; doi:10.3390/molecules30214179)
Supplement: Supplementary file 1 [file molecules-30-04179-s001.zip › molecules-3933229-supplementary.pdf]

Article

GC/MS Analysis, Cytotoxicity, and Antimicrobial Properties of Six Moroccan Essential Oils Traditionally Used for COVID-19 Prevention

Houda Zaher[1,2*], José Francisco Quílez del Moral[2*], Sanae Lemrabet[3], Azucena Gonzalez-Coloma[4*], Bouchaib Bencharki[1]

1. Laboratory of Agro Alimentary and Health, Faculty of Sciences and Techniques, Hassan First University of Settat, B.P. 539, Settat 26000, Morocco; bouchaib.bencharki@uhp.ac.ma
2. Department of Organic Chemistry, Institute of Biotechnology, University of Granada, 18071 Granada, Spain
3. Virology Department, National Institute of Hygiene, Ministry of Health, B.P. 769, Rabat 10020, Morocco; sanae.lem@gmail.com
4. Institute of Agricultural Sciences, Spanish National Research Council (CSIC), 28006 Madrid, Spain

* Correspondence: zaherhouda@gmail.com (H.Z.); jfquilez@ugr.es (J.F.Q.d.M.); azu@ica.csic.es (A.G.-C.)

Supplementary Materials

(1) A. absinthium

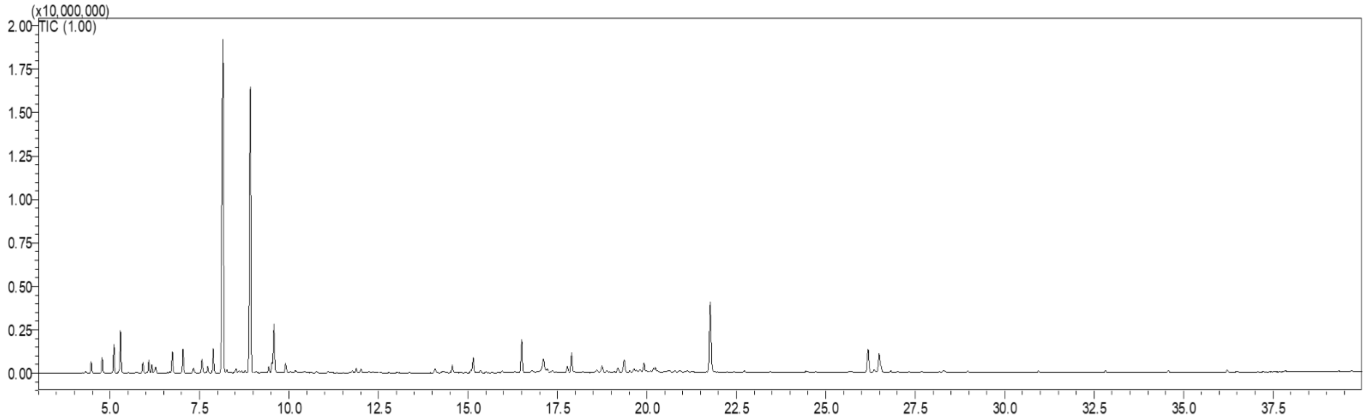

(2) E. globulus

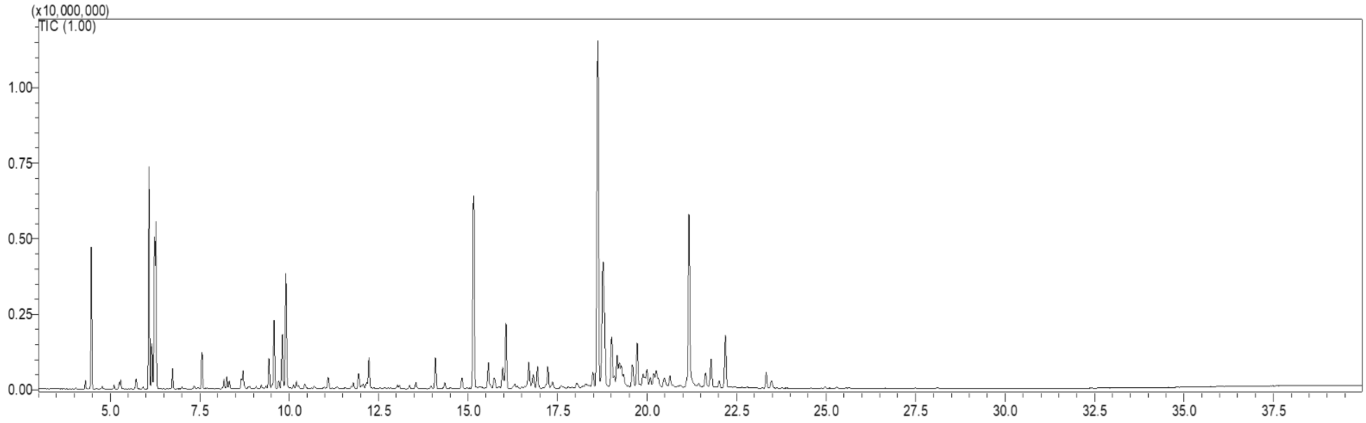

(3) T. vulgaris

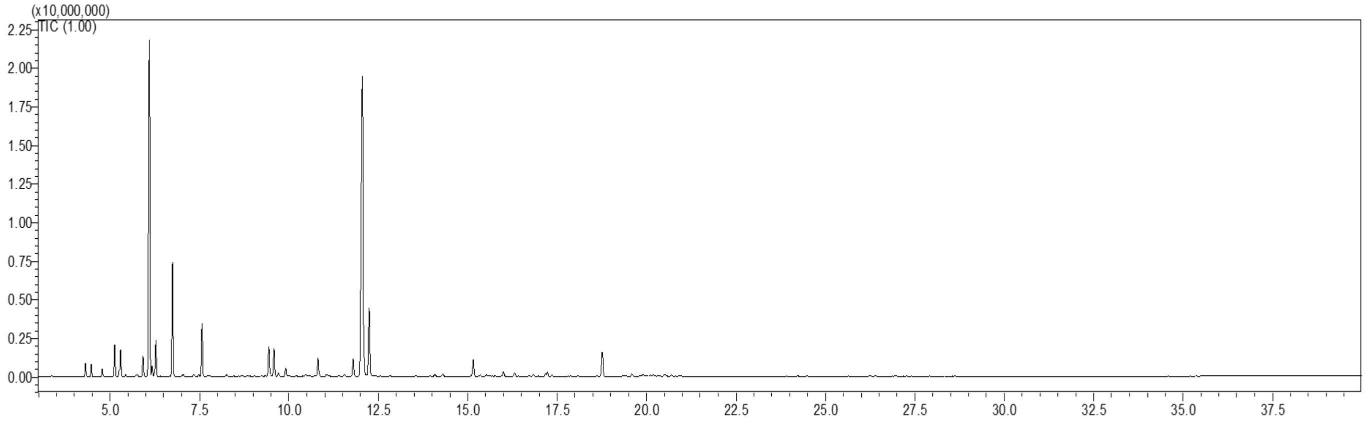

(4) S. aromaticum

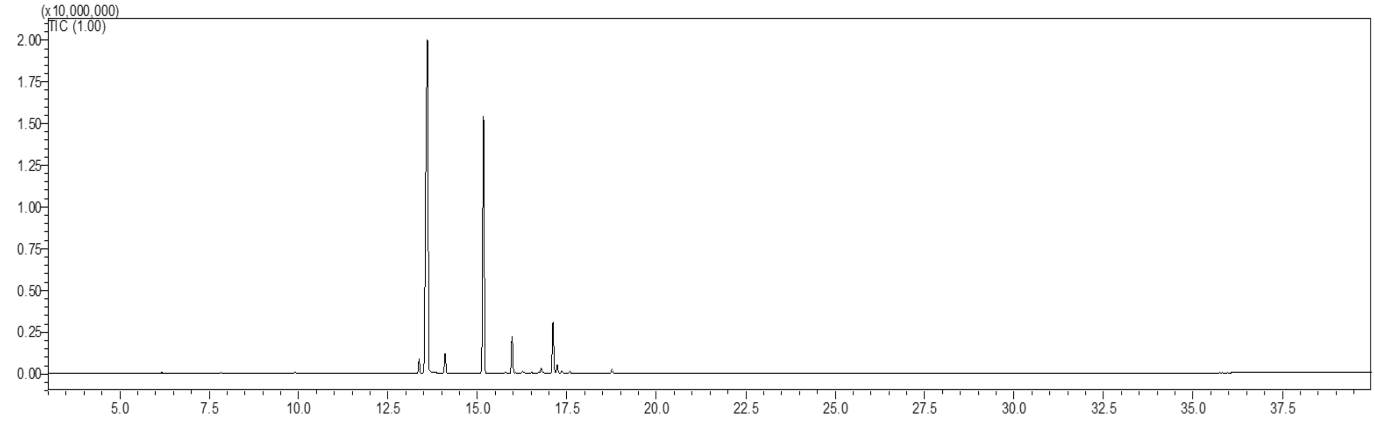

(5) A. herba-alba

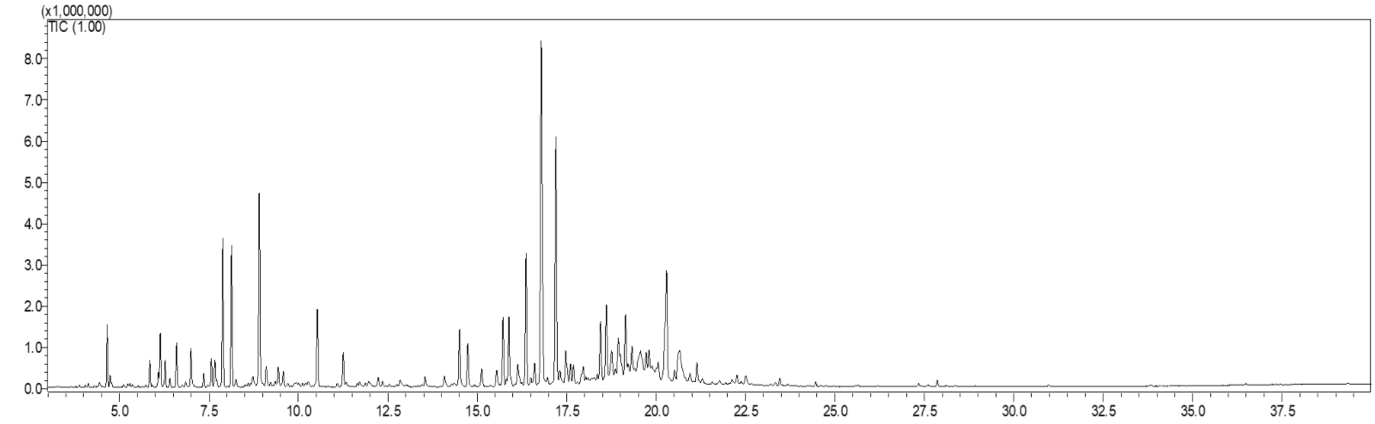

(6) S. chamaecyparisses

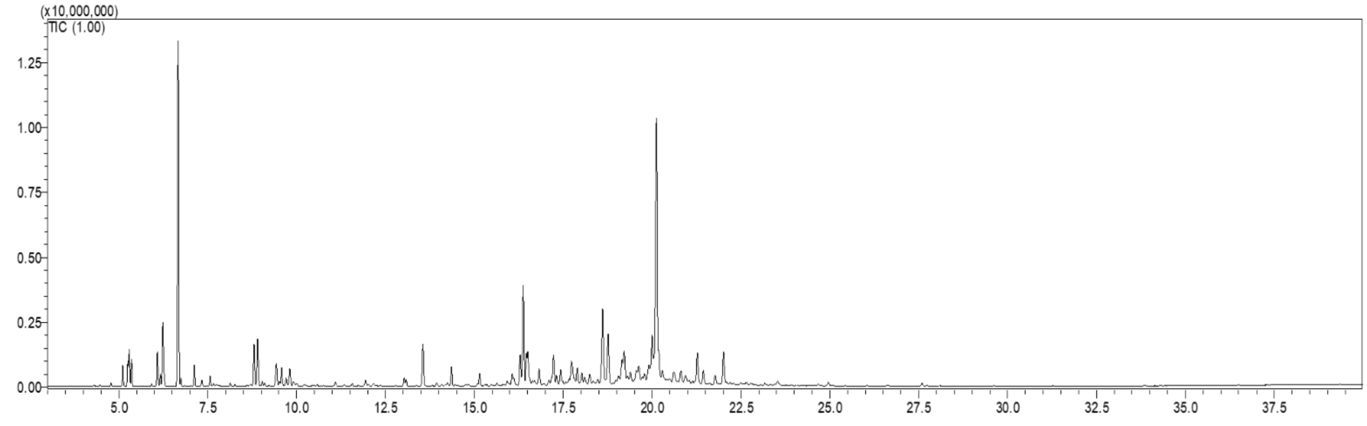

Figure S1. Gas chromatography chromatograms of the essential oils extracted from the following plants: **(1)** *A. absinthium L.*; **(2)** *E. globulus*; **(3)** *T. vulgaris*; **(4)** *S. aromaticum*; **(5)** *A. herba-alba*; and **(6)** *S. chamaecyparisses*.

Table S1. Chemical composition of *Artemisia absinthium*

Ret. Time	% Area	Compound	% similarity
**4,470**	0.64	.alpha.-pipene	
**4,779**	0.88	Camphene	
**5,109**	1.64	Sabinene	
**5,291**	2.58	.beta.-Myrcene	
**5,916**	0.64	.ALPHA. TERPINENE	
**6,083**	0.74	PARA CYMENE	
**6,166**	0.48	LIMONENE	
**6,273**	0.41	1,8-Cineole	
**6,740**	1.32	.gamma.-Terpinene	
**7,038**	1.54	trans Sabinene hydrate	
**7,331**	0.30	TERPINOLENE	
**7,566**	1.06	Linalool	
**7,727**	0.43	trans Sabinene hydrate	
**7,883**	1.67	.BETA.-THUJONE	
**8,153**	29.02	.ALPHA.-THUJONE	
**8,262**	0.20	1-TERPINEOL	
**8,519**	0.26	3-Thujanol, (1S,3S,4S,5R)-(+)-	
**8,600**	0.16	Sabinyl acetate	
**8,670**	0.12	1-TERPINEOL	
**8,771**	0.15	(1R)-cis-Verbenol	
**8,920**	24.34	CAMPHOR	
**9,433**	0.45	L-Borneol	
**9,523**	0.63	(-)-cis-Myrtanol	
**9,578**	3.68	(-)-4-Terpineol	
**9,903**	0.75	.ALPHA. TERPINEOL	
**10,176**	0.17	5-Heptenoic acid, 2,6-dimethyl-2-vinyl-	79
**11,878**	0.31	METHYLBENZOATE	
**12,013**	0.27	Thymol	
**14,083**	0.32	.alpha.-Copaene	
**14,563**	0.54	METHYL EUGENOL	
**15,080**	0.20	.beta.-Ylangene	
**15,147**	1.23	Caryophyllene	
**15,351**	0.15	(1R,2S,6S,7S,8S)-8-Isopropyl-1-methyl-3-methylenetricyclo[4.4.0.02,7]decane-rel-	
**15,955**	0.15	.ALPHA.-HUMULENE	
**16,504**	2.65	GERMACRENE-D	
**17,111**	1.52	3,6-Dihydrochamazulene	
**17,222**	0.25	.gamma.-Muurolene	

**17,362**	0.12	3,6-Dihydrochamazulene	84
**17,781**	0.55	Cyclohexane, 1-methyl-2,4-bis(1-methylethenyl)-, (1.alpha.,2.beta.,4.beta.)-	86
**17,898**	1.53	Elemol	
**18,606**	0.14	Spathulenol	87
**18,750**	0.59	CARYOPHYLLENE OXIDE	
**19,192**	0.39	Cycloheptane, 4-methylene-1-methyl-2-(2-methyl-1-propen-1-yl)-1-vinyl-	87
**19,366**	1.21	3,6-Dihydrochamazulene	86
**19,642**	0.29	Benzene, (2-ethyl-4-methyl-1,3-pentadienyl)-, (E)-	69
**19,723**	0.15	.gamma.-Eudesmol	89
**19,806**	0.20	.alpha.-longipinene	79
**19,922**	0.70	.alpha.-Patchoulene	81
**20,177**	0.27	.alpha.-Cadinol	75
**20,237**	0.24	.beta.-Eudesmol	90
**20,787**	0.13	3-(p-Ethylphenyl)adamantanol-1	60
**21,772**	6.92	Chamazulene	
**26,180**	2.18	Arborescin	84
**26,359**	0.21	(1R,4R,5S)-1,8-Dimethyl-4-(prop-1-en-2-yl)spiro[4.5]dec-7-ene	86
**26,491**	2.14	geranyl-.alpha.-terpinene	88

Table S2. Chemical composition of *Eucalypthus globulus*

Ret. Time	% Area	Compound	% similarity
**4,311**	0.2	alpha.-Thujene	
**4,473**	3.6	d-.alpha.-Pinene	
**5,250**	0.18	.DELTA.3-Carene	
**5,293**	0.21	.BETA.-MYRCENE	
**5,727**	0.25	.alpha.-phellandrene	
**6,086**	6.29	p-Cymene	
**6,169**	1.26	LIMONENE	
**6,241**	4.2	.beta.-Phellandrene	
**6,277**	4.52	1,8-Cineole	
**6,743**	0.58	.gamma.-Terpinene	
**7,008**	0.06	Linalool oxide	
**7,344**	0.09	trans-Ocimenol	
**7,567**	1.02	Linalool	
**8,181**	0.26	Fenchol	
**8,261**	0.34	(E)-p-2-Menthen-1-ol	
**8,332**	0.22	alpha.-Campholenal	
**8,663**	0.29	p-menth-2-en-1-ol	87
**8,713**	0.61	Sabinyl acetate	88
**9,436**	0.97	ENDO-BORNEOL	
**9,580**	2.33	4-TERPINEOL	
**9,710**	0.25	p-Cymen-8-ol	
**9,810**	1.82	Crypton	
**9,909**	3.95	.ALPHA. TERPINEOL	
**11,091**	0.36	p-Cumic aldehyde	
**11,941**	0.51	phellandral	
**12,232**	0.91	Carvacrol	
**13,024**	0.13	Bicycloelemene	
**13,085**	0.12	.delta.-Elemene	
**13,362**	0.12	.alpha.-Copaene	83
**13,541**	0.24	m-Eugenol	
**13,963**	0.1	.alpha.-amorphene	88
**14,088**	1.08	Copaene	
**14,348**	0.23	.beta.-elemene	
**14,827**	0.37	.alpha.-Gurjunene	
**15,154**	7.33	trans-Caryophyllene	
**15,568**	0.86	(+)-AROMADENDRENE	
**15,727**	0.5	Bicyclo[4.3.0]nonane, 4,5-dimethyl-1-(1-hydroxy-2-propyl)-5-(3-methyl-2-pentenoyl)-	65
**15,967**	0.94	.ALPHA.-HUMULENE	
**16,059**	2.39	NEOALLOOCIMENE	
**16,697**	0.89	.beta.-Guaiene	80
**16,820**	0.49	VALENCENE	88
**16,939**	0.81	.beta.-Bisabolene	89

**17,228**	0.67	.DELTA.-CADINENE	
**18,487**	0.5	(-)-Globulol	
**18,624**	15	Spathulenol	87
**18,773**	7.67	CARYOPHYLLENE OXIDE	
**19,012**	2.23	Viridiflorol	
**19,093**	0.32	geranyl-.alpha.-terpinene	77
**19,164**	1.14	Isoleptospermone	89
**19,228**	1.08	cis-Eudesm-6-en-11-ol	82
**19,283**	0.63	Isospathulenol	83
**19,592**	1.03	cis-Eudesm-6-en-11-ol	83
**19,726**	1.59	Aromadendrane-4,10-diol	87
**19,883**	0.79	Alloaromadendrene	85
**19,998**	0.58	Torreyol	83
**20,089**	0.32	Arctiol	81
**20,183**	0.59	.alpha.-Cadinol	88
**20,258**	0.68	.beta.-Eudesmol	69
**20,327**	0.19	Neointermedeol	82
**20,641**	0.37	Ledene	77
**21,172**	7.52	Farnesol	
**21,632**	0.51	trans, trans-Farnesal	
**21,791**	1.18	Valerenal	86
**22,014**	0.21	(2E,6E,10E)-3,7,11,15-Tetramethylhexadeca-2,6,10,14-tetraen-1-yl formate	
**22,188**	2.15	(1S,4R,5S)-1-Methyl-4-(prop-1-en-2-yl)spiro[4.5]dec-7-ene-8-carbaldehyde	87
**23,331**	0.51	FARNESYL ACETATE 3	
**23,478**	0.28	Pentafluorobenzoic acid, tridec-2-ynyl ester	70

Table S3. Chemical composition of *thymus vulgaris*

Ret. Time	% Area	Compound	% similarity
**4,311**	0.73	.alpha.-Thujene	
**4,473**	0.68	.alpha.-pipene	
**4,782**	0.41	Camphene	
**5,127**	1.78	Amyl vinyl carbinol	
**5,294**	1.82	.beta.-Myrcene	
**5,433**	0.12	3-Octanol	
**5,772**	0.16	3-Carene	
**5,920**	1.28	.ALPHA. TERPINENE	
**6,099**	25.87	p-Cymene	
**6,172**	0.62	LIMONENE	
**6,279**	2.39	1,8-Cineole	
**6,746**	7.21	.gamma.-Terpinene	
**7,041**	0.11	trans Sabinene hydrate	
**7,340**	0.16	(+)-4-Carene	
**7,479**	0.13	Cymenene	
**7,568**	3.34	Linalool	
**8,263**	0.12	1-TERPINEOL	
**9,438**	2.26	L-Borneol	
**9,582**	2.03	4-TERPINEOL	
**9,708**	0.23	p-Cymen-8-ol	
**9,909**	0.62	.ALPHA. TERPINEOL	
**10,812**	1.41	Isothymol methyl ether	
**11,547**	0.18	Citral	
**11,795**	1.34	Thymol	
**12,053**	33.33	Thymol	
**12,246**	5.23	Carvacrol	
**14,081**	0.17	.alpha.-Copaene	83
**14,305**	0.22	.BETA. BOURBONENE	
**15,155**	1.44	Caryophyllene	
**15,353**	0.17	GERMACRENE-D	87
**15,999**	0.42	Butanoic acid, 2-methyl-, 3,7-dimethyl-2,6-octadienyl ester, (E)-	
**16,313**	0.27	.gamma.-Muurolene	94
**16,825**	0.16	.alpha.-amorphene	84
**17,170**	0.23	.gamma.-Cadinene	87
**17,230**	0.31	.DELTA.-CADINENE	
**17,357**	0.12	cis-Calamenene	
**18,761**	2.19	CARYOPHYLLENE OXIDE	
**19,590**	0.24	epi-.gamma.-Eudesmol	

**19,895**	0.14	.gamma.-Muurolene	90
**20,499**	0.25	(-)-Globulol	84
**20,695**	0.11	.ALPHA.-BISABOLOL	

Table S4. Chemical composition of *Syzgium aromaticum*

Ret. Time	% Area	Compound	% similarity
**9,900**	0.17	Methyl salicylate	
**13,369**	1.34	.alpha.-Cubebene	
**13,604**	54.96	Eugenol	
**14,099**	1.9	.alpha.-Copaene	
**15,172**	29.18	trans-Caryophyllene	
**15,787**	0.14	Naphthalene, 1,2,3,4,4a,5-hexahydro-4,7-dimethyl-1-(1-methylethyl)-, (1S,4S,4aS)	
**15,968**	3.79	.ALPHA.-HUMULENE	
**16,066**	0.09	NEOALLOOCIMENE	
**16,317**	0.08	.alpha.-amorphene	
**16,511**	0.15	GERMACRENE-D	
**16,783**	0.63	Farnesene	
**17,115**	5.41	Eugenol acetate	
**17,232**	0.9	.DELTA.-CADINENE	
**17,363**	0.27	cis-Calamenene	
**17,586**	0.22	Naphthalene, 1,2,3,4,4a,7-hexahydro-1,6-dimethyl-4-(1-methylethyl)-	
**18,760**	0.49	CARYOPHYLLENE OXIDE	

Table S5. Chemical composition of *artemisia helba-alba*

Ret. Time	% Area	Compound	% similarity
**4,653**	1.41	Cyclohexanebutanal, 2-methyl-3-oxo-, cis-	83
**4,733**	0.28	4,4-DIMETHYLBUT-2-ENOLIDE	
**5,292**	0.06	.beta.-Myrcene	
**5,845**	0.67	1,8-Cineole	83
**6,085**	0.38	PARA CYMENE	
**6,140**	1.41	Decane, 2-cyclohexyl-	82
**6,275**	0.66	1,8-Cineole	
**6,406**	0.24	2(3H)-Furanone, 5-ethenyldihydro-5-methyl- (CAS) 4-Methyl-4-vinylbutyrolactone	
**6,594**	1.17	cis-Arbusculone	
**6,996**	1.07	cis-Arbusculone	
**7,351**	0.36	Ethyl 2-(5-methyl-5-vinyltetrahydrofuran-2-yl)propan-2-yl carbonate	
**7,565**	0.75	Linalool	

**7,665**	0.77	HOTRIENOL	
**7,884**	4.31	.BETA.-THUJONE	
**8,132**	4.09	Thujone	
**8,256**	0.27	ISOPHORONE	81
**8,726**	0.22	p-Mentha-1(7),8(10)-dien-9-ol	85
**8,905**	6.02	(+)-2-Bornanone	
**9,100**	0.60	cis-Chrysanthenol	
**9,435**	0.55	L-Borneol	
**9,579**	0.42	4-TERPINEOL	
**10,530**	2.19	Nordavanone	
**11,254**	0.97	cis-Chrysanthenol acetate	
**12,233**	0.26	Carvacrol	
**13,543**	0.30	Isoeugenol	81
**14,086**	0.40	Copaene	
**14,504**	1.97	CIS JASMONE	
**14,735**	1.31	davana furan	87
**15,124**	0.57	3aH-Cyclopentacycloocten-3a-ol, 1,2,3,4,7,8,9,9a-octahydro-1,5,8,8-tetramethyl-, [1R-(1.alpha.,3a.beta.,9a.alpha.)]-	76
**15,544**	0.58	(1RS,5RS,6RS,7RS)-2,2,6,7-tetramethyl-10-oxatricyclo[[5.2.1.0(!,6)]decan-5-ol	74
**15,720**	2.13	2-Butyl-5-methyl-3-(2-methylprop-2-enyl)cyclohexanone	76
**15,810**	0.21	2-Butyl-5-methyl-3-(2-methylprop-2-enyl)cyclohexanone	75
**15,883**	2.49	2-Butyl-5-methyl-3-(2-methylprop-2-enyl)cyclohexanone	77
**16,135**	0.86	3aH-Cyclopentacycloocten-3a-ol, 1,2,3,4,7,8,9,9a-octahydro-1,5,8,8-tetramethyl-, [1R-(1.alpha.,3a.beta.,9a.alpha.)]-	75
**16,363**	4.21	DAVANA ETHER 1	
**16,501**	0.25	GERMACRENE-D	
**16,606**	0.81	2-Butyl-5-methyl-3-(2-methylprop-2-enyl)cyclohexanone	77
**16,789**	14.48	DAVANA ETHER 1	
**16,962**	0.22	(2R,2'S,5S,5'S)-2,5'-Dimethyl-5-(prop-1-en-2-yl)-5'-vinylhexahydro-[2,2'-bifuran]-3(2H)-one	
**17,197**	8.86	**Davana ether**	
**17,313**	0.40	(2R,2'S,5S,5'S)-2,5'-Dimethyl-5-(prop-1-en-2-yl)-5'-vinylhexahydro-[2,2'-bifuran]-3(2H)-one	
**17,474**	1.46	Artedouglasia oxide C	
**17,607**	0.63	(2R,2'S,5S,5'S)-2,5'-Dimethyl-5-(prop-1-en-2-yl)-5'-vinylhexahydro-[2,2'-bifuran]-3(2H)-one	
**17,691**	0.60	(2R,2'S,5S,5'S)-2,5'-Dimethyl-5-(prop-1-en-2-yl)-5'-vinylhexahydro-[2,2'-bifuran]-3(2H)-one	
**17,903**	0.29	2-Butenal, 2-methyl-4-(2,6,6-trimethyl-1-cyclohexen-1-yl)-	67
**17,965**	0.51	(S)-2,6,6-Trimethyl-2-((2S,5R)-5-methyl-5-vinyltetrahydrofuran-2-yl)-2H-pyran-3(6H)-one	89
**18,356**	0.19	(2R,2'S,5S,5'S)-2,5'-Dimethyl-5-(prop-1-en-2-yl)-5'-vinylhexahydro-[2,2'-bifuran]-3(2H)-one	
**18,448**	2.18	Davanone	
**18,610**	2.91	1H-Cycloprop[e]azulen-7-ol, decahydro-1,1,7-trimethyl-4-methylene-, [1ar-(1a.alpha.,4a.alpha.,7.beta.,7a.beta.,7b.alpha.)]-	88
**18,755**	1.36	CARYOPHYLLENE OXIDE	

**18,944**	1.50	1-((1R,2R,3R)-2-(3-Isopropylfuran-2-yl)-3-methylcyclopentyl)ethanone	69
**19,146**	2.10	3-Methyl-but-2-enoic acid, 1,7,7-trimethyl-bicyclo[2.2.1]hept-2-yl ester	86
**19,326**	0.84	Humulenol-II	87
**19,523**	0.76	(S,Z)-5-Hydroxy-6-methyl-2-((2S,5R)-5-methyl-5-vinyltetrahydrofuran-2-yl)hepta-4,6-dien-3-one	72
**19,564**	1.00	Humulenol-II	80
**19,800**	0.63	(S,Z)-5-Hydroxy-6-methyl-2-((2S,5R)-5-methyl-5-vinyltetrahydrofuran-2-yl)hepta-4,6-dien-3-one	76
**20,291**	5.58	2-Propenal, 3-(2,6,6-trimethyl-1-cyclohexen-1-yl)-	82
**20,519**	0.42	Oxalic acid, butyl 1-menthyl ester	75
**20,656**	3.21	Davanone	86
**21,143**	0.66	cis-Hydroxydavanone	
**22,264**	0.29	Artemisyl acetate	
**23,462**	0.24	Hexahydrofarnesyl acetone	
**27,864**	0.19	Eicosane	

Table S6. Chemical composition of *Santolina chamaecyparisses*

Ret. Time	% Area	Compound	% similarity
**4,472**	0.04	.alpha.-PINENE	
**4,781**	0.09	Camphene	
**5,111**	0.73	Sabinene	
**5,255**	0.86	Sabinene	
**5,293**	1.28	.BETA.-MYRCENE	
**5,354**	0.91	yomogi alcohol	
**6,085**	1.3	PARA CYMENE	
**6,169**	0.44	LIMONENE	
**6,239**	2.73	.beta.-Phellandrene	
**6,669**	15.58	Artemisia ketone	
**6,746**	0.32	.gamma.-Terpinene	
**7,124**	0.81	ARTEMISIA ALCOHOL	
**7,334**	0.24	.ALPHA.-TERPINOLENE	
**7,566**	0.38	Linalool	
**8,803**	1.67	cis-Chrysanthemyl alcohol	
**8,905**	2.11	(+)-2-Bornanone	
**9,031**	0.22	Lavandulol	
**9,432**	1.17	endo-Borneol	
**9,518**	0.19	1,8-menthadien-4-ol	85
**9,578**	0.79	4-TERPINEOL	
**9,708**	0.35	p-Cymen-8-ol	
**9,809**	0.79	Cryptone	
**11,093**	0.13	p-Cumic aldehyde	

**11,942**	0.25	Phellandral	89
**13,026**	0.38	Elixene	
**13,086**	0.28	.delta.-Elemene	
**13,552**	1.96	.alpha.-Longipinene	
**14,355**	0.88	.BETA. ELEMENE	
**15,150**	0.48	Caryophyllene	
**16,062**	0.51	NEOALLOOCIMENE	86
**16,117**	0.25	2H-Cycloprop[c]indene-2,3(3ah)-dione, hexahydro-3a,7,7-trimethyl-	77
**16,288**	1.55	.gamma.-Curcumene	
**16,376**	4.81	.alpha.-Curcumene	
**16,464**	1.4	n-Pentadecanol	
**16,505**	1.79	GERMACRENE-D	
**16,635**	0.52	(2R,3R,6S)-6-Isopropyl-3-methyl-2-(prop-1-en-2-yl)-3-vinylcyclohexanone	81
**16,822**	0.9	(1S,2E,6E,10R)-3,7,11,11-Tetramethylbicyclo[8.1.0]undeca-2,6-diene	91
**17,100**	0.32	(2S,3S,6S)-6-Isopropyl-3-methyl-2-(prop-1-en-2-yl)-3-vinylcyclohexanone	91
**17,167**	0.37	(1R,2S,6S,7S,8S)-8-Isopropyl-1-methyl-3-methylenetricyclo[4.4.0.02,7]decane-rel-	87
**17,228**	1.52	.DELTA.-CADINENE	
**17,312**	0.48	d-Nerolidol	84
**17,426**	0.81	NEROLIDOL	78
**17,735**	1.49	CARYOPHYLLENE OXIDE	83
**17,817**	0.16	9-Methoxycalamenene	67
**17,895**	0.79	9,12-Octadecadienal, dimethyl acetal (CAS)	78
**18,022**	0.5	NEROLIDOL	
**18,100**	0.4	Longifolenaldehyde	81
**18,244**	0.52	LONGIFOLENALDEHYDE	77
**18,477**	0.17	Di-epi-.alpha.-cedrene-(I)	81
**18,611**	4.41	Spathulenol	87
**18,763**	3.19	Epizizanone	85
**18,967**	0.2	Salvial-4(14)-en-1-one	86
**19,060**	0.4	Vulgarone A	80
**19,157**	1.46	.beta.-Oplopenone	85
**19,216**	1.82	(+)-TRANS-CARAN-CIS-3-OL	79
**19,299**	0.37	Isolongifolene	83
**19,389**	0.59	Ylangenol	77
**19,553**	0.53	2(3H)-Naphthalenone, 4,4a,5,6,7,8-hexahydro-4a,5-dimethyl-3-(1-methylethylidene)-, (4ar-cis)-	80
**19,624**	0.83	Isospathulenol	86
**19,713**	0.18	Isospathulenol	76
**19,787**	0.3	(2R,3R,4aR,5S)-2-Hydroxy-4a,5-dimethyl-3-(prop-1-en-2-yl)-3,4,4a,5,6,7-hexahydronaphthalen-1(2H)-one	78
**19,903**	0.92	.delta.-Cadinene	88

**19,998**	2.77	(2R,3R,4aR,5S,8aS)-2-Hydroxy-4a,5-dimethyl-3-(prop-1-en-2-yl)-2,3,4,4a,5,6-hexahydronaphthalen-1(8aH)-one	79
**20,123**	18.15	Longiverbenone	
**20,289**	0.62	((4aS,8S,8aR)-8-Isopropyl-5-methyl-3,4,4a,7,8,8a-hexahydronaphthalen-2-yl)methanol	82
**20,608**	0.68	Muurola-4,10(14)-dien-1.beta.-ol	78
**20,810**	0.7	(1R,7S,E)-7-Isopropyl-4,10-dimethylenecyclodec-5-enol	85
**20,935**	0.57	(1R,7S,E)-7-Isopropyl-4,10-dimethylenecyclodec-5-enol	82
**21,042**	0.18	trans-Sesquisabinene hydrate	79
**21,274**	1.61	((4aS,8S,8aR)-8-Isopropyl-5-methyl-3,4,4a,7,8,8a-hexahydronaphthalen-2-yl)methanol	82
**21,441**	0.72	6-Isopropenyl-4,8a-dimethyl-1,2,3,5,6,7,8,8a-octahydro-naphthalen-2-ol	84
**21,775**	0.47	Cycloheptane, 4-methylene-1-methyl-2-(2-methyl-1-propen-1-yl)-1-vinyl-	81
**22,009**	1.69	Elixene	83
